# Harlequin Syndrome Following Regional Liposomal Bupivacaine Use in a Partial Sternectomy

**DOI:** 10.7759/cureus.28005

**Published:** 2022-08-14

**Authors:** Delaney A Dalldorf, Alexandria Hart, Stuart A Grant, Emily G Teeter

**Affiliations:** 1 Department of Anesthesiology, University of North Carolina at Chapel Hill, Chapel Hill, USA; 2 Anesthesiology, Baylor St. Lukes Medical Center, Houston, USA

**Keywords:** ultrasound-guided erector spinae plane block (espb), harlequin syndrome, liposomal bupivacaine, general thoracic surgery, regional anesthesiology

## Abstract

Harlequin syndrome is a condition in which disruption of the autonomic nervous system results in ipsilateral anhidrosis and pallor of the face. We report the first documented case of Harlequin syndrome following the use of liposomal bupivacaine, in which a patient developed symptoms five hours after a bilateral erector spinae plane (ESP) block with liposomal bupivacaine before partial sternectomy. It is additionally unique as the first report of delayed onset of symptoms. The proposed mechanism is the diffusion of the anesthetic into the paravertebral space with cephalad migration to the T2-T3 level, where facial vasomotor fibers exit the spinal cord.

## Introduction

Harlequin syndrome is a rare condition characterized by unilateral facial flushing and sweating [1]. Although the exact mechanism remains unclear, it is thought to be due to a disruption in the autonomic nervous system. Specifically, lesions near T2 or T3 can disrupt the stellate ganglion and superior cervical ganglion, which would result in ipsilateral anhidrosis and skin pallor of the face, neck, and upper chest. Despite its alarming presentation, this syndrome appears to be benign and self-limiting when caused by a regional anesthetic technique [2]. Though there have been isolated case reports of Harlequin syndrome following various regional anesthetic techniques, there are no documented reports of this condition presenting after the use of liposomal bupivacaine.

Liposomal bupivacaine, first approved in 2011 for bunionectomies and hemorrhoidectomies, is a long-acting local anesthetic used to produce postsurgical analgesia [3]. The FDA issued expanded use to include surgical infiltration in 2015, allowing the use of liposomal bupivacaine for transversus abdominus plane (TAP) blocks. It has since been used to provide analgesia in thoracic, orthopedic, and abdominal surgeries, as well as off-label use in peripheral nerve blocks [4]. Liposomal bupivacaine is becoming increasingly popular for preventing post-thoracotomy pain syndrome [5]. This case report describes the first documented case of Harlequin syndrome following a bilateral erector spinae plane block with liposomal bupivacaine. The patient gave consent for the publication of this case report. This manuscript adheres to the applicable Enhancing the QUAlity and Transparency Of health Research (EQUATOR) guideline.

## Case presentation

**Figure 1:**
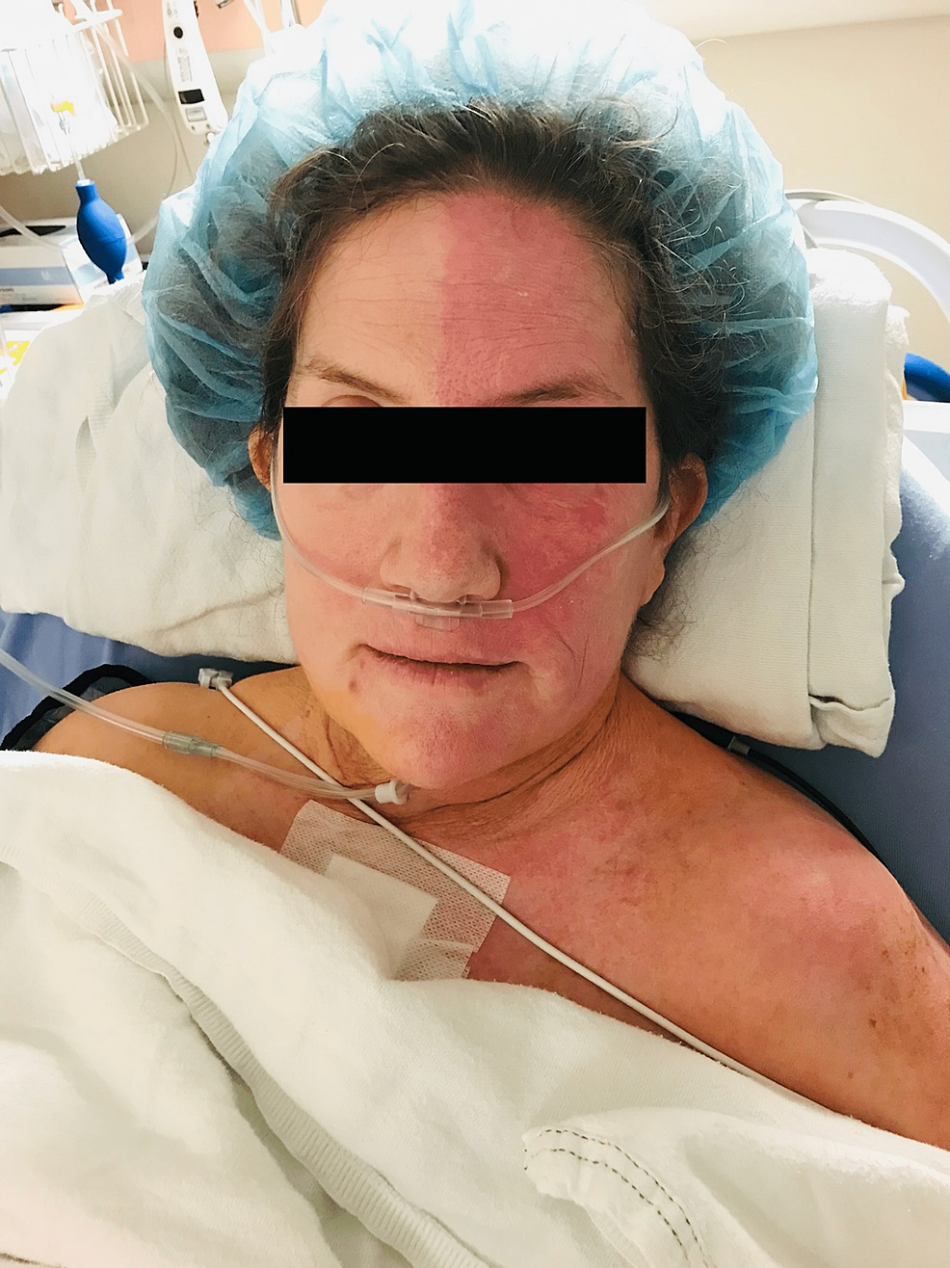
Unilateral facial flushing. The image was taken 30 minutes after arrival at PACU

A 52-year-old, 77kg female with metastatic breast cancer, anxiety, and chronic pain presented with a destructive metastatic lesion in the upper body of the manubrium. She was scheduled to undergo a partial sternectomy with reconstruction. A pre-operative regional anesthetic was planned as part of a multimodal approach. The regional anesthesia team evaluated the patient before the surgery and elected to proceed with bilateral erector spine plane (ESP) blocks at the T2 level, given the location of the lesion in the upper body of the manubrium. She was premedicated with midazolam 1 milligram and fentanyl 50 micrograms. A sterile ultrasound-guided technique was used, and 10 ml (133mg) of liposomal bupivacaine with 20mL of 0.25% bupivacaine for a total volume of 30 ml was injected on each side. The procedure was uneventful other than difficulty visualizing the needle during injection on the left side. The patient was then taken to the operating room, where she underwent a standard general endotracheal anesthetic. There were no complications during the surgical procedure, and she was extubated postoperatively and taken to the post-anesthesia care unit. No facial flushing was present during the case or at extubation. She was assessed by the attending anesthesiologist 15 minutes after her arrival in the post-anesthesia care unit, who noted the new onset of unilateral facial erythema and diaphoresis (Figure 1).

The onset of symptoms was approximately five hours after ESP injection. She was hemodynamically stable and there were no signs of Horner’s syndrome such as miosis or ptosis. The patient was counseled regarding expectant management of her symptoms with the possibility of delayed resolution of symptoms given the use of liposomal bupivacaine in the block. By the time of the anesthesia team’s postoperative visit the following morning, all symptoms had resolved. The patient was discharged without recurrence of any signs of Harlequin syndrome.

## Discussion

Harlequin syndrome has previously been described following regional anesthetic techniques, including thoracic paravertebral, erector spinae plane, and interscalene brachial plexus blocks, as well as thoracic and obstetric lumbar epidurals [1-9]. All previous cases of harlequin syndrome have occurred immediately following injection of plain local anesthetic. While ours is not the first reported case of Harlequin syndrome associated with an ESP block, it is unique because this is the first reported case of the delayed onset of Harlequin syndrome because of the use of liposomal bupivacaine.

Liposomal bupivacaine’s novel property as an extended-release local anesthetic with a duration of approximately 72 hours makes it quite useful in regional anesthesia, including fascial plane blocks. One such fascial plane block, the ESP block, was first described in 2016 and involves the injection of local anesthetic in the paraspinal fascial plane, deep to the erector spinae muscle and superficial to the thoracic transverse process [10]. The ESP block is popular due to relatively few contraindications and the opportunity for significant cranial-caudal spread from a single injection point. The mechanism of action of the ESP block has been debated but is currently believed to be the result of diffusion of local anesthetic anteriorly to the ventral and dorsal rami of spinal nerves [11]. The ESP block effectively replicates the effects of a paravertebral block while minimizing the risk of pleural injury.

The development of Harlequin syndrome after ESP block in this patient is hypothesized to be due to diffusion of local anesthetic into the paravertebral space with cephalad migration and activity where facial vasomotor fibers exit the spinal cord near the T2-T3 level. The presence of unilateral symptoms may be related to poor needle visualization resulting in incorrect local anesthetic deposition deep to the ESP on one side. When Harlequin syndrome is associated with a regional anesthetic technique, the onset of symptoms typically occurs within an hour following the procedure. Our case presents a unique timeline, with a longer than expected symptom onset observed five hours after the ESP block. We believe this delayed onset is related to liposomal bupivacaine’s extended-release formulation.

Fortunately, management of Harlequin syndrome related to a regional anesthetic consists mainly of watchful waiting and reassurance, with symptoms typically resolving over 6-12 hours without any permanent nerve dysfunction. It is important to note the potential for delayed onset and prolonged duration of Harlequin syndrome when liposomal bupivacaine is used.

## Conclusions

This case report highlights an important adverse effect related to an increasingly common regional anesthetic technique, the ESP block. It also calls attention to a novel change with longer than expected symptom onset and prolonged duration of side effects related to the use of a relatively new local anesthetic, liposomal bupivacaine. Fully understanding the implications of liposomal bupivacaine use and the prolonged timeline of onset and resolution of effect is important. Harlequin Syndrome is managed with watchful waiting and reassurance.
